# First Nanoparticles of a Conductor Based on the Organic Donor Molecule BETS: κ-(BETS)_2_FeCl_4_

**DOI:** 10.3390/ma14164444

**Published:** 2021-08-08

**Authors:** Kane Jacob, Christophe Faulmann, Dominique de Caro, Lydie Valade

**Affiliations:** 1 LCC-CNRS (Laboratoire de Chimie de Coordination), Université de Toulouse, CNRS, UPS, F-31077 Toulouse, France; kane.jacob@lcc-toulouse.fr (K.J.); lydie.valade@lcc-toulouse.fr (L.V.); 2 CEMES-CNRS (Centre D’Elaboration de Matériaux et D’Etudes Structurales), Université de Toulouse, CNRS, UPS, F-31055 Toulouse, France

**Keywords:** bis(ethylenedithio)tetraselenafulvalene BETS, molecular conductors, electrocrystallization technique, nanoparticles, amphiphilic imine

## Abstract

Nanoparticles of the molecular superconductor (BETS)_2_FeCl_4_ were obtained by the electrochemical oxidation of BETS in the presence of [(C_2_H_5_)_4_N]FeCl_4_ and an amphiphilic imine (OATM), acting as a growth controlling agent. When the reaction was carried out with a molar ratio OATM/BETS of 10, roughly spherical nanoparticles exhibiting sizes in the 10–40 nm range were observed. X-ray diffraction patterns evidenced the growth of (BETS)_2_FeCl_4_ nanoparticles with the κ-type structure. The current-voltage characteristic recorded on an individual nanoparticle aggregate was fitted with a Shockley diode model. A saturation current of 1216 pA and a threshold voltage of 0.62 V were extracted from this model. This latter value was consistent with roughly half of the energy gap of the semiconducting nano-crystalline aggregate.

## 1. Introduction

**Figure 1:**
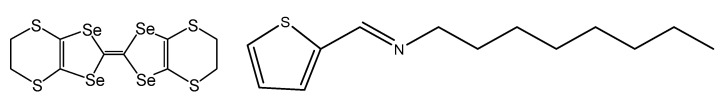
Formulas for BETS (**left**) and OATM (**right**).

The interest for nanoparticles of tetrathiafulvalene (TTF)-based organic conductors and superconductors began in the mid-1990s. One of the main motivations was the fabrication of a new generation of electronic devices based on molecular components. In addition, controlling the size of organic conductors at the nanoscale level presented an important opportunity to explore potential new physical properties. In 1994, Ward et al. reported that the brief exposure of (111)-oriented Au surfaces to TTF‧TCNQ (TCNQ: tetracyanoquinodimethane) led to the formation of TTF‧TCNQ nanoclusters, as evidenced by scanning tunneling microscopy (average thickness of 0.15 nm) [1]. A longer exposure resulted in the formation of highly oriented TTF‧TCNQ needles, with their *b* axis perpendicular to the substrate. Moreover, the oxidation of BEDT-TTF (bis(ethylenedithio)tetrathiafulvalene) in the presence of I_3_^−^ on freshly cleaved highly oriented pyrolytic graphite led to monolayers of β-(BEDT-TTF)_2_I_3_ [2]. These monolayers formed by aggregation of two-dimensional clusters whose height was 1.55 nm. Furthermore, Rovira et al. reported the selective deposition of a TTF compound bearing two carboxylate groups onto predefined nanoscale domains of a silicon chip with an accuracy of 40 nm [3]. In 2001, Jeszka et al. reported the preparation of (BEDT-TTF)_2_I_3_ nanocrystals (sizes in the 80–200-nm range) using the rapid solidification of a *para*-dichlorobenzene solution of (BEDT-TTF)_2_I_3_ with the subsequent sublimation of the solvent [4]. Finally, spherical nanoparticles of a mixed valence conductor containing a metal-bis(dithiolene) complex, i.e., [(CH_3_)_4_N][Ni(dmit)_2_]_2_ (dmit^2−^: 2-thioxo-1,3-dithiole-4,5-dithiolato), were prepared by template chemistry [5]. The electrochemical growth of [(CH_3_)_4_N][Ni(dmit)_2_]_2_ as nanoparticle chains was performed within the pores of an anodic aluminum oxide film [5]. For 10 years, our group has been working on the chemical or electrochemical synthesis of nanoparticles of molecule-based conductors and superconductors. Unlike some methods described above, we were able to obtain nanoparticles on a scale of at least 50 mg, which represented a significant amount for TTF-based materials. Common to our synthetic routes was the use of a growth controlling agent which prevented undesired morphologies, such as elongated platelets, needles or wires. The main families of growth controllers that we have explored were imidazolium-based ionic liquids, long alkyl chain quaternary ammonium salts, oligomers and neutral amphiphilic molecules [6,7]. Until now, we have successfully prepared nanoparticles of three ambient pressure organic superconductors, i.e., (TMTSF)_2_ClO_4_ [8,9] (TMTSF: tetramethyltetraselenafulvalene), (BEDT-TTF)_2_I_3_ [10,11] and (BEDT-TTF)_2_Cu(NCS)_2_ [12,13]. In the present communication, we report on the preparation of the first nanoparticles based on the organic donor molecule BETS, i.e., bis(ethylenedithio)tetraselenafulvalene (Figure 1).

## 2. Materials and Methods

Tetrahydrofurane (THF), ethanol and chlorobenzene were distilled under argon before use. BETS [14], [(C_2_H_5_)_4_N]FeCl_4_ [15] and 1-octanamine, *N*-(2-thienylmethylene) OATM (Figure 1) [16] were prepared according to previously described procedures.

Elemental analyses were performed by the Microanalysis Service of LCC-CNRS. Infrared spectra were taken at room temperature (in KBr matrix) on a Perkin Elmer Spectrum GX spectrophotometer (PerkinElmer, Villebon-sur-Yvette, France). X-ray diffraction patterns were recorded at room temperature on a PANalytical X’Pert Pro (Theta-Theta) diffractometer (Malvern Pananalytical, Palaiseau, France) with Cu K_α1_, K_α2_ radiation (*λ* = 1.54059, 1.54442 Å). For transmission electron microscopy (TEM, JEOL Europe SAS, Croissy, France), the samples were sonicated in ether and placed onto a holey carbon-copper grid. TEM experiments were performed on a JEOL Model JEM 1011 operating at 100 kV. AFM topographic images and current-voltage (*I*–*V*) curves were acquired on an AFM Smarts SPM 1000 (AIST-NT, HORIBA Scientific, Palaiseau, France) in conductivity mode using Au-coated cantilever tips (PPP-NCL Au-10 from Nanosensors, resonance frequency: 146–236 kHz, force constant: 21–98 N.m^−1^, tip radius: ~10 nm). About 1 mg of the nanoparticle powder was dispersed in diethyl ether (0.5 mL). A drop of this dispersion was placed onto a gold substrate (0.7 × 0.7 cm^2^) previously cleaned with acetone, water, and carefully dried.

The synthesis of κ-(BETS)_2_FeCl_4_ nanoparticles was performed in a one-compartment electrocrystallization cell equipped with two platinum wire electrodes (length *L* = 1 cm, diameter *d* = 1 mm). The cell was filled with BETS (10 mg, 0.018 mmol), [(C_2_H_5_)_4_N]FeCl_4_ (20 mg, 0.061 mmol), OATM (10 or 20 molar eq. vs. BETS) and THF (40 mL). The electrolysis was conducted at 60 °C under potentiostatic conditions (0.85 V vs. SCE) for 6 h. The solution was stirred during the entire electrolysis procedure. The air-stable black powder of κ-(BETS)_2_FeCl_4_ (about 10 mg) was collected by filtration, washed with THF, and finally dried. Elemental analysis (C_20_H_16_Cl_4_FeS_8_Se_8_), calculated: C 17.9%; H 1.2%; S 19.1%, found C 17.3%; H 0.7%; S 18.6%.

## 3. Results and Discussion

In the early 1990s, the BETS molecule proved to be a good candidate for obtaining molecule-based superconductors. Among organic superconductors based on BETS, needle crystals of (BETS)_2_MX_4_ (M = Fe, Ga, In; X = Cl, Br) have a λ-type structure, whereas plate crystals have a κ-type structure [17]. (BETS)_2_GaCl_4_ with the λ-type structure undergoes a superconducting transition at 5.5 K under ambient pressure, whereas λ-(BETS)_2_FeCl_4_ undergoes a metal-to-insulator transition at 8.3 K. By applying a pressure of 2.5 kbar, the antiferromagnetic insulating state of λ-(BETS)_2_FeCl_4_ is suppressed and this compound becomes a superconductor with a critical temperature of about 2 K [17]. Finally, (BETS)_2_FeCl_4_ with the κ-type structure undergoes a superconducting transition at 0.1 K under ambient pressure [17]. To investigate the superconducting state at the nanoscale level, Hassanien et al. prepared single layers of (BETS)_2_GaCl_4_ molecules on Ag(111) surfaces, using an ultra-high vacuum technique [18]. They reported that a superconducting gap had still been detected in just four pairs of (BETS)_2_GaCl_4_ molecules.

**Figure 2:**
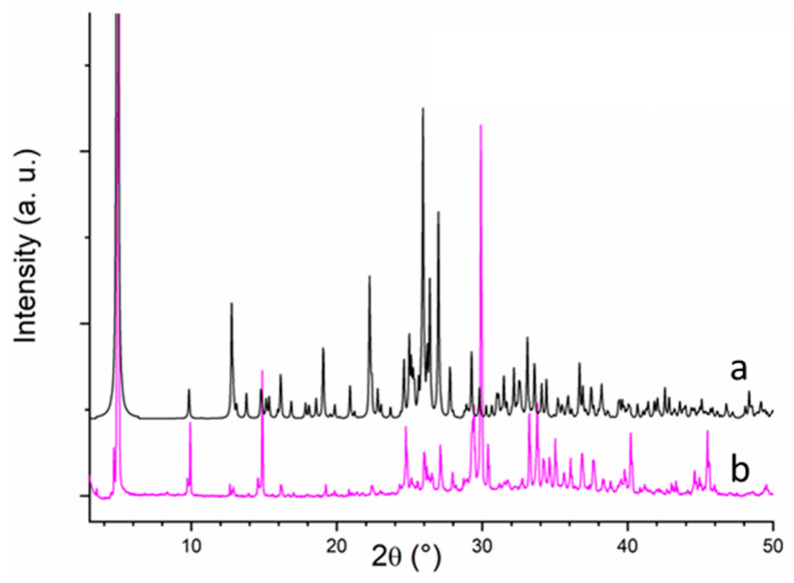
X-ray diffraction pattern for κ-(BETS)_2_FeCl_4_: (**a**) simulated from single crystals; (**b**) nanoparticles grown in the presence of OATM (10 molar eq. vs. BETS).

The work reported in this communication mainly focuses on the preparation and morphological characterization of the first nanoparticles of a BETS-based molecular superconductor, i.e., κ-(BETS)_2_FeCl_4_. We have recently described the synthesis of nanoparticles of the κ-(BEDT-TTF)_2_I_3_ superconductor for which the growth controlling agent was the neutral OATM amphiphilic molecule [11]. This molecule was especially interesting because it afforded various possibilities of interactions with the BEDT-TTF molecule, in favor of a better control of the nanoparticle size (π–π interactions and S···S van der Waals interactions) and a better control of their dispersion (long octyl chain). These nanoparticles exhibited an average size of about 25 nm. As reported by Kobayashi et al. in 1996, the electrochemical oxidation of BETS on a Pt wire at a constant current in the presence of the inorganic anion FeCl_4_^−^ led to a mixture of λ-(BETS)_2_FeCl_4_ and κ-(BETS)_2_FeCl_4_ [19]. However, when the electrochemical oxidation was conducted under constant voltage conditions, black plate crystals of κ-(BETS)_2_FeCl_4_ were selectively grown (dimension: ~0.40 × 0.35 × 0.02 mm^3^) [20]. We have then considered the electrochemical growth of (BETS)_2_FeCl_4_ in an organic medium containing the amphiphilic OATM species at a constant voltage. In [20], crystals of κ-(BETS)_2_FeCl_4_ were prepared from a 5–10% ethanol/chlorobenzene solution containing BETS and [(C_2_H_5_)_4_N]FeCl_4_ (4.0 V vs. SCE). By using the same experimental conditions and further adding OATM to the solution, no product was obtained on the platinum electrode. In contrast, the electrochemical oxidation of BETS in a tetrahydrofurane solution of [(C_2_H_5_)_4_N]FeCl_4_ and OATM at 0.85 V vs. SCE at 60 °C for 6 h led to a black solid which dropped from the electrode due to the constant agitation of the solution. X-ray diffraction patterns recorded at room temperature were in agreement with the κ-(BETS)_2_FeCl_4_ phase (Figure 2) [21] (see Supplementary Materials). Figure 2a showed the X-ray diffraction pattern simulated from κ-(BETS)_2_FeCl_4_ single crystals whereas Figure 2b showed that recorded for nanoparticles grown in the presence of 10 molar eq. of OATM vs. BETS. That for nanoparticles prepared in the presence of 20 molar eq. of OATM vs. BETS was very similar.

**Figure 3:**
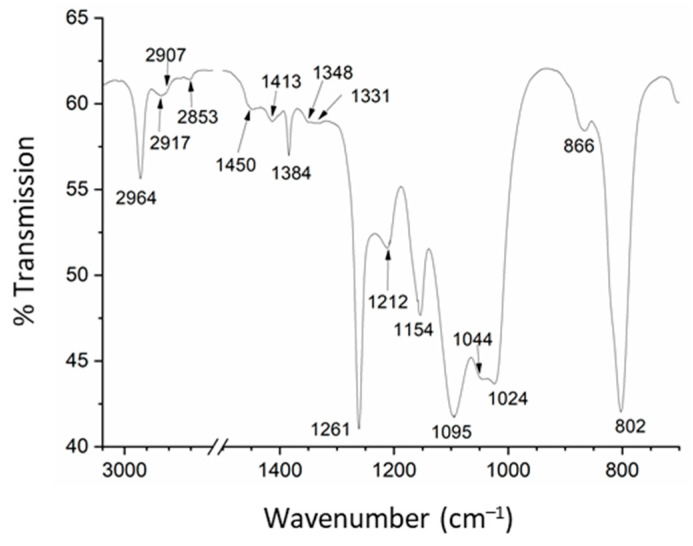
Infrared spectrum for κ-(BETS)_2_FeCl_4_ nanoparticles grown in the presence of OATM.

Infrared spectra for nanoparticles were close to that described for a κ-(BETS)_2_FeCl_4_ single crystal (Figure 3) [22]. The two bands at 2964 (m) and 2917 (w) cm^−1^ were assigned to CH stretching modes for methylene CH_2_ groups. The weak band at 1450 cm^−1^ was attributed to a C=C stretching mode coupled to intramolecular electron oscillations [22]. By comparison with the BEDT-TTF molecule [23], the vibration modes at 1261 (s), 1212 (m) and 1154 (m) cm^−1^ were assigned to CH_2_ out-of-plane vibrations. Finally, the peaks at 823 (sh) and 802 (s) cm^−1^ were assigned to the stretching modes for C–S and C–Se groups, respectively [22].

**Figure 4:**
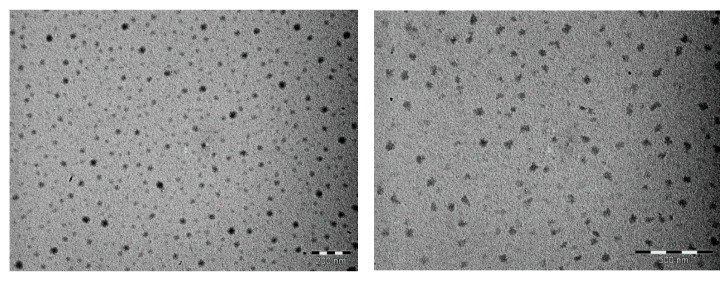
Electron micrographs for κ-(BETS)_2_FeCl_4_ nanoparticles (**left**: 10 molar eq. of OATM vs. BETS, bar = 200 nm; **right**: 20 molar eq. of OATM vs. BETS, bar = 500 nm).

For a molar ratio OATM/BETS of 10, electron micrographs evidenced roughly spherical nanoparticles exhibiting sizes in the 10–40 nm range (Figure 4). For a molar ratio OATM/BETS of 20, TEM images showed irregularly shaped nanoparticles (sizes in the 10–70 nm range, Figure 4). Obtaining nanoparticles instead of plate single crystals showed that OATM played a crucial role in the control of the growth of κ-(BETS)_2_FeCl_4_. We assumed that π–π stacking occurred between the thiophene group of OATM and the C_3_Se_2_ ring of the BETS molecule in addition to S···S van der Waals interactions between the S atom of OATM and those of BETS. Furthermore, OATM molecules could adsorb onto the Pt electrode surface. Therefore, the germination process of κ-(BETS)_2_FeCl_4_ was more closely controlled at the electrode-solution interface, and the growth process was quickly blocked due to the steric hindrance of C_8_ chains, leading to well-dispersed nanoparticles.

**Figure 5:**
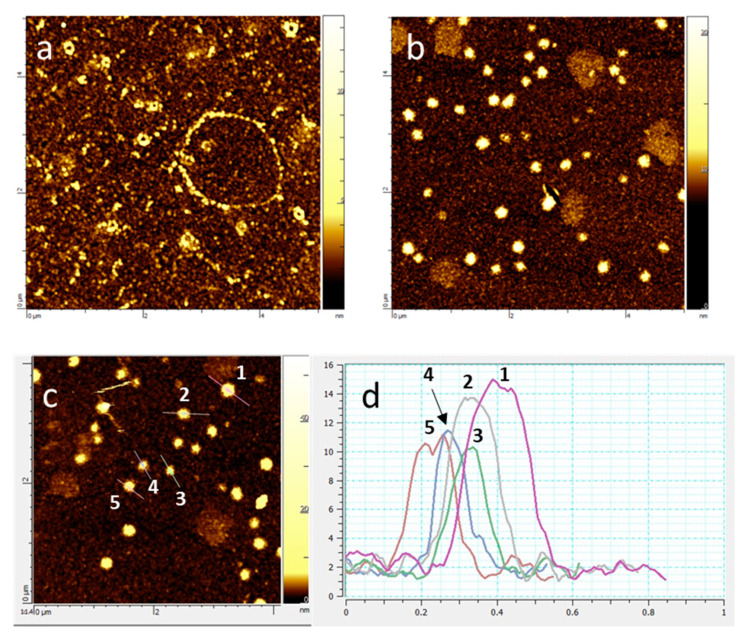
AFM topographic images for κ-(BETS)_2_FeCl_4_ nanoparticles: (**a**) 10 molar eq. of OATM vs. BETS, scan size: 5 × 5 μm^2^; (**b**) 20 molar eq. of OATM vs. BETS, scan size: 5 × 5 μm^2^. (**c**) Five selected nanoparticles (20 molar eq. of OATM vs. BETS) for size determination and (**d**) graph showing the line profile analysis where the Y axis is the height (diameter) of the nanoparticles and the X axis is the length of the line scanned by the tip.

Topographic images confirmed that κ-(BETS)_2_FeCl_4_ was grown as nano-objects, as shown on Figure 5. For a molar ratio OATM/BETS of 10 (Figure 5a), particles tended to aggregate or to form circles onto the gold substrate, whereas they remained fairly well dispersed for a molar ratio of 20 (Figure 5b). In the latter case, the line profile analysis of AFM topographic images (Figure 5c,d) evidenced that particles exhibited diameters in the 10–15 nm range, in agreement with the size of the smaller nanoparticles observed by TEM.

**Figure 6:**
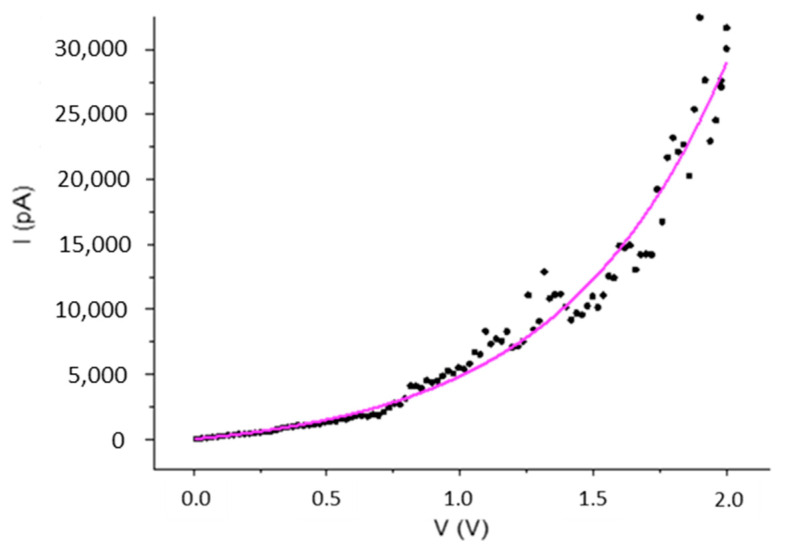
*I‒V* characteristic for a nanoparticle aggregate prepared in the presence of 10 molar eq. of OATM vs. BETS and least-squares fit of the region corresponding to positive bias voltages.

*I*–*V* curves recorded at room temperature for a nanoparticle aggregate (molar ratio OATM/BETS of 10) were asymmetric in shape (see Supplementary Materials). The nanoparticle aggregate exhibited a characteristic semiconductor-like behavior related to various possible boundaries, i.e., nanoparticle-nanoparticle, tip-nanoparticle and substrate-nanoparticle. A similar semiconducting behavior (at room temperature) has previously been observed on a nanoparticle aggregate of (BEDT-TTF)_2_I_3_ grown in the presence of OATM [10]. From the linear part of the curve around the origin of the coordinate system, we evaluated an energy gap of about 1.30 eV (see Supplementary Materials). A least-squares fit using the Shockley diode equation, $I=I_{0}\;\left[e^{\frac{V}{V_{0}}}-1\right]$ where *I*_0_ represented the saturation current and *V*_0_ the threshold voltage (energy barrier) [10], was performed in the region corresponding to positive bias voltages (Figure 6). From the fit, we extracted *I*_0_ = 1216 ± 142 pA and *V*_0_ = 0.62 ± 0.02 V. The activation energy barrier obtained from the fit (0.62 eV) was consistent with half of the energy gap (1.30/2 = 0.65 eV). Nanoparticles prepared for a molar ratio OATM/BETS of 20 were unstable during the curve recording, and no usable *I*–*V* characteristic was obtained.

## 4. Conclusions

In this communication, we have described the first nanoparticles of a BETS-based superconductor, i.e., κ-(BETS)_2_FeCl_4_. The growth controlling agent evaluated in this study, namely an amphiphilic imine, prevented the preparation of (BETS)_2_FeCl_4_ as micro-sized platelets or needles. This strategy, based on the use of a molecule bearing a heterocycle and a long alkyl chain and which has previously shown its efficiency for growing nano-objects of TMTSF- and BEDT-TTF-based superconductors [8,9,10,11,12,13], was applied successfully here with BETS. We are currently exploring alternative experimental conditions to obtain nanoparticles of (BETS)_2_FeCl_4_ with the λ-type structure. Indeed, this phase is much more attractive than the κ phase, in particular as regards the physical properties.

## Data Availability

The authors confirm that the data supporting the findings of this study are available within this communication.

## Supplementary Materials

The following are available online at https://www.mdpi.com/article/10.3390/ma14164444/s1, Refined cell and indexed peaks for κ-(BETS)_2_FeCl_4_ nanoparticles grown in the presence of OATM (10 molar eq. vs. BETS) and *I‒V* curve for a κ-(BETS)_2_FeCl_4_ nanoparticle aggregate grown in the presence of OATM (10 molar eq. vs. BETS).
