# Supplementary material for: First Nanoparticles of a Conductor Based on the Organic Donor Molecule BETS: κ-(BETS)2FeCl4
Source: Materials (Basel). 2021 Aug 8;14(16):4444. doi: 10.3390/ma14164444 (PMC8398930; doi:10.3390/ma14164444)
Supplement: Supplementary file 1 [file materials-14-04444-s001.zip › materials-1303121-supplementary.pdf]

Supplementary Material

First nanoparticles of a conductor based on the organic donor molecule BETS: κ-$(BETS)_2FeCl_4$

Kane Jacob, Christophe Faulmann, Dominique de Caro and Lydie Valade (LCC-CNRS, Toulouse, France)

1) Refined cell and indexed peaks for κ-$(BETS)_2FeCl_4$ nanoparticles grown in the presence of OATM (10 molar eq. *vs.* BETS)

Bibliographic data:	
Audit creation date:	1996-08-14
Audit creation method:	CSD-ConQuest-V1
Author name:	H.Kobayashi, H.Tomita, T.Naito, A.Kobayashi, F.Sakai, T.Watanabe, P.Cassoux
Chemical name systematic:	bis(bis(Ethylenedithio)tetraselenafulvalene) tetrachloro-iron
Formula sum:	$C_{20}H_{16}Cl_4Fe_1S_8Se_8$
Journal name:	J.Amer.Chem.Soc.
Journal volume:	118
Page first:	368
Structure and profile data:	
Formula sum:	$Fe_4Cl_{24}Se_{32}S_{32}C_{80}$
Formula mass/ g/mol:	5587.7800
Density (calculated)/ g/cm^3	2.6044
F(000):	2592,0000
Mass Absorption Coefficient/ cm^2/g:	82.6906
Space group (No.):	P n m a (62)
Lattice parameters:	
a/ Å:	11.680(4)
b/ Å:	35.92(2)
c/ Å:	8.491(3)
alpha/ °:	90
beta/ °:	90
gamma/ °:	90
V/ 10^6 pm^3	3562.2200

Non-indexed peaks

No.	Th. (o) [°]	d-sp. (o) [Å]	Rel. Int. [%]	Height [cts]	Sin2 (o)
1	4.6790	18.870510	3.32	9948.6450	167

Indexed peaks

No.	h	k	l	2Θ (c) [°]	2Θ (o) [°]	2Θ (d) [°]	d-sp. (c) [Å]	d-sp. (o) [Å]	d-sp. (d) [Å]
1	0	2	0	4.9251	4.9249	0.0002	17.928120	17.928700	-0.000582
2	0	4	0	9.8592	9.9326	-0.0733	8.964062	8.898049	0.066012
3	0	3	1	12.7915	12.6421	0.1494	6.915005	6.996370	-0.081365
4	1	0	1	12.8874	12.9014	-0.0140	6.863772	6.856346	0.007426
5	0	6	0	14.8118	14.5743	0.2375	5.976040	6.072887	-0.096846
6	1	3	1	14.8717	14.8794	-0.0077	5.952113	5.949033	0.003080
7	0	5	1	16.1754	16.1322	0.0432	5.475216	5.489779	-0.014563
8	2	2	1	19.0769	19.2390	-0.1620	4.648482	4.609694	0.038787
9	1	1	2	22.4286	22.3939	0.0347	3.960833	3.966892	-0.006059
10	1	4	2	24.4240	24.3144	0.1096	3.641562	3.657730	-0.016169
11	0	10	0	24.8111	24.7688	0.0423	3.585625	3.591647	-0.006022
12	2	1	2	26.0621	26.0029	0.0592	3.416274	3.423919	-0.007645
13	2	9	0	27.0617	27.1027	-0.0410	3.292314	3.287432	0.004883
14	0	11	1	29.3313	29.4326	-0.1014	3.042522	3.032273	0.010249
15	0	12	0	29.8786	29.8933	-0.0146	2.988020	2.986590	0.001430
16	1	11	1	30.3310	30.4082	-0.0773	2.944482	2.937173	0.007309
17	0	1	3	31.7358	31.7744	-0.0386	2.817270	2.813932	0.003337
18	4	3	1	33.2470	33.2214	0.0256	2.692586	2.694606	-0.002020
19	3	9	1	33.8385	33.7772	0.0613	2.646862	2.651525	-0.004663
20	0	13	1	34.1576	34.2230	-0.0654	2.622860	2.618001	0.004859

21	3	6	2	34.6525	34.6113	0.0412	2.586526	2.589509	-0.002983
22	0	14	0	35.0067	35.0107	-0.0040	2.561160	2.560875	0.000285
23	2	2	3	35.6102	35.6226	-0.0124	2.519124	2.518278	0.000846
24	2	3	3	36.0619	36.0701	-0.0082	2.488602	2.488059	0.000544
25	4	7	1	36.9278	36.8612	0.0666	2.432213	2.436453	-0.004240
26	4	2	2	37.6836	37.6433	0.0403	2.385150	2.387612	-0.002462
27	2	14	0	38.3377	38.3177	0.0201	2.345944	2.347127	-0.001183
28	4	4	2	38.7099	38.8123	-0.1024	2.324241	2.318344	0.005897
29	1	9	3	39.8289	39.7924	0.0365	2.261483	2.263475	-0.001993
30	0	16	0	40.2083	40.2002	0.0081	2.241015	2.241447	-0.000431
31	0	14	2	41.1454	41.1607	-0.0153	2.192116	2.191334	0.000781
32	1	11	3	43.0264	43.0129	0.0136	2.100534	2.101165	-0.000631
33	1	0	4	43.3518	43.3140	0.0378	2.085522	2.087254	-0.001732
34	4	12	1	44.6262	44.6040	0.0222	2.028877	2.029836	-0.000960
35	1	17	1	44.9233	44.9218	0.0015	2.016146	2.016211	-0.000065
36	2	0	4	45.4834	45.4794	0.0040	1.992611	1.992779	-0.000168
37	3	3	4	49.5027	49.5363	-0.0336	1.839822	1.838653	0.001169
38	6	10	0	53.4699	53.4762	-0.0063	1.712293	1.712105	0.000188
39	2	12	4	55.3753	55.3957	-0.0203	1.657800	1.657239	0.000560
40	7	1	1	56.1317	56.1246	0.0072	1.637240	1.637432	-0.000192
41	4	19	0	58.1352	58.1254	0.0098	1.585495	1.585739	-0.000244
42	2	18	3	58.8269	58.8270	0.0000	1.568490	1.568490	0.000001
43	3	20	2	60.8807	60.8801	0.0006	1.520405	1.520418	-0.000014
44	4	16	3	61.5598	61.5449	0.0149	1.505250	1.505578	-0.000329
45	8	1	0	63.6657	63.6817	-0.0159	1.460440	1.460113	0.000327
46	4	4	5	64.3036	64.3085	-0.0049	1.447483	1.447385	0.000098
47	7	13	1	66.2833	66.2850	-0.0017	1.408970	1.408938	0.000032

2) *I-V* curve for a κ-$(BETS)_2FeCl_4$ nanoparticle aggregate grown in the presence of OATM (10 molar eq. *vs.* BETS)

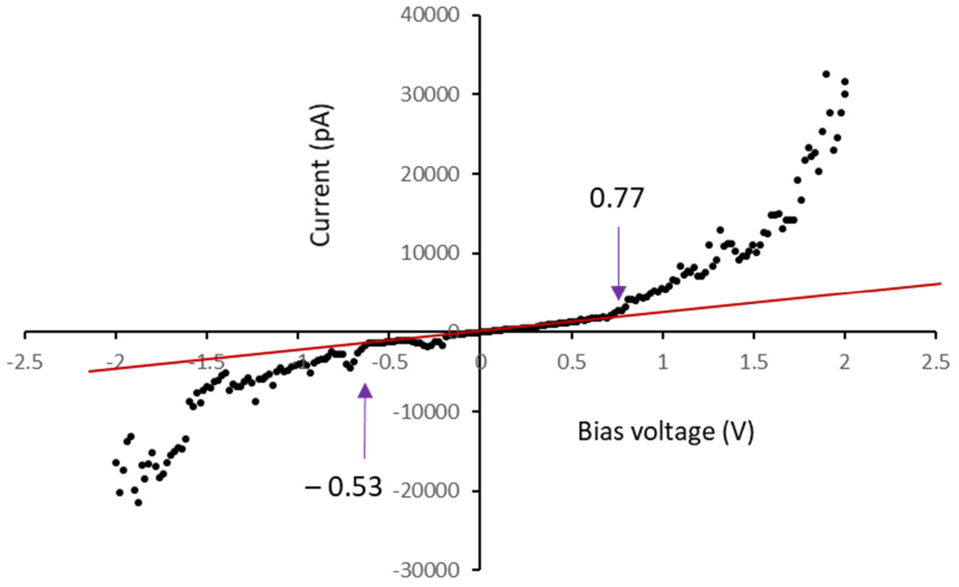

From the linear part of the curve around the origin of the coordinate system, we determine ΔV = 0.77 – (–0.53) = 1.30 V, *i.e.*, an energy gap of E_g = 1.30 eV.
